# SEARCHBreast: An online resource designed to increase the efficiency of using materials derived from breast cancer studies in animals

**DOI:** 10.1002/path.4755

**Published:** 2016-08-22

**Authors:** Valerie Speirs, Bethny Morrisey, Ingunn Holen, Karen Blyth, Phil Carter, Claude Chelala, Louise Jones

**Affiliations:** ^1^ Leeds Institute of Cancer and PathologyUniversity of LeedsLeedsUK; ^2^ Academic Unit of Clinical OncologyUniversity of SheffieldSheffieldUK; ^3^ Cancer Research UK Beatson InstituteGlasgowUK; ^4^ Barts Cancer InstituteLondonUK

Sir,

We applaud the recent Journal of Pathology Annual Review Issue on Models of Human Disease. This highlighted the need to identify robust and relevant models to address human disease, recognising their strengths and limitations 1, at the same time being cognisant of employing the principles of the 3Rs (Replacement, Reduction, and Refinement) 2 in research, and improving experimental reproducibility by adequately reporting on pathology data gathered from animal tissues, by introducing the MINPEPA guidelines (minimum information for publication of experimental pathology data 3.

As a corollary to these excellent articles contained within the Annual Review 1, 3 we would like to draw attention to a new resource we have developed which is designed to facilitate sharing of archival animal material in breast cancer, also encouraging scientists to consider alternative models by developing 3D *in vitro* models using human clinical material. Called SEARCHBreast (**S**haring **E**xperimental **A**nimal **R**esources: **C**oordinating **H**oldings – Breast), this is a virtual online resource. SEARCHBreast allows researchers who may have surplus animal materials which they may be willing to share collaboratively to upload these to the SEARCHBreast website (https://searchbreast.org). Researchers wishing to obtain such models can register, allowing access to well‐characterised materials which have been previously generated from animal studies (e.g. transgenic, xenograft, patient‐derived xenograft (PDX)) thereby reducing or even eliminating the need to initiate new *in vivo* experiments 4, 5.

As well as making previously hidden archival animal materials more widely available, SEARCHBreast also provides information to improve the use of animals in research e.g. links to online technical information or standard operating procedures which give guidelines on experimental procedures in animals. SEARCHBreast is also developing a bioinformatics pipeline, designed to take advantage of the large amount of data being generated through various ‘omics’ technologies. This aims to allow researchers to select models that are the most relevant to address their biological question. Additionally, SEARCHBreast encourages scientists to consider alternative humanised *in vitro* models of breast cancer 6, through engagement with the Breast Cancer Now Tissue Bank cell culture programme 7.

We encourage your readers to explore this resource; following a simple on line registration process, academic researchers have free access to SEARCHBreast at https://searchbreast.org.

## Author Contributions

KB, CC, LJ, IH, VS conceived of the study, secured funding and participated in its design and coordination and helped to draft the manuscript. BM participated in the design of the study, management of the resource and prepared the first draft; PC participated in the design of the study and constructed the database and website. All authors read and approved the final manuscript.


Valerie Speirs,^1*^ Bethny Morrisey,^1^ Ingunn Holen,^2^ Karen Blyth,^3^ Phil Carter,^4^ Claude Chelala^4^ and Louise Jones^4^
*^1^Leeds Institute of Cancer and Pathology, University of Leeds, Leeds, UK*
*^2^Academic Unit of Clinical Oncology, University of Sheffield, Sheffield, UK*
*^3^Cancer Research UK Beatson Institute, Glasgow, UK*
*^4^Barts Cancer Institute, London, UK*
*Correspondence to: Prof V Speirs, E‐mail: v.speirs@leeds.ac.uk
*The authors declare no conflict of interest*.
